# Circadian aspects of mortality in hospitalized patients: A retrospective observation from a large cohort

**DOI:** 10.1002/nop2.1711

**Published:** 2023-03-08

**Authors:** Miao Fang, Shunju Xiang, Xiaoqiang Xiao, Qianning Mo, Yang Si

**Affiliations:** ^1^ Department of Neurology Sichuan Academy of Medical Science & Sichuan Provincial People's Hospital Chengdu China; ^2^ Department of Anesthesiology, West China Hospital Sichuan University Chengdu China; ^3^ West China School of Nursing Sichuan University Chengdu China; ^4^ Sichuan Provincial Center for Mental Health, The Center of Psychosomatic Medicine of Sichuan Provincial People's Hospital University of Electronic Science and Technology of China Chengdu China; ^5^ Department of Medical Administration Sichuan Academy of Medical Science & Sichuan Provincial People's Hospital Chengdu China; ^6^ University of Electronic Science and Technology of China Chengdu China

**Keywords:** circadian rhythm, death, inpatients, mortality, nursing care

## Abstract

**Aim:**

This study aimed to describe the circadian characteristics of hospitalized mortality in order to provide nursing guidance for preventing in‐hospital mortality.

**Design:**

A retrospective analysis on inpatient information was implemented.

**Methods:**

Harmonic Analysis of Time Series was applied to quantify the periodic structure of the frequency of the occurrence of death.

**Results:**

A total of 3300 cases were included in the present study (male, 63.4% and median age 73 years), including 1540 (46.7%) ICU patients. Incidence of overall hospitalized death exhibited a circadian pattern, presenting peaks from 07:00 to 12:00 and 15:00 to 20:00 P.M., with 21.5% and 13.1% increase above the average at those peak points, respectively. Similarly, the incidence of sudden cardiac death (SCD) showed peaks between 06:00–12:00 and 15:00–20:00, with a 34.7% and 28.0% increase above the average at peak time, respectively. The distribution of death incidence revealed no statistical difference between SCD and non‐SCD (*p* = 0.525).

## 1 INTRODUCTION

Accumulating evidence has indicated the importance of circadian variations on regulating body functions (e.g., sleep–wake cycle, hormone secretion, blood and temperature regulation, and autonomic nervous tone; Baschieri & Cortelli, 2019; Crnko et al., 2019; Kvaslerud et al., 2010) which plays a great role on disease development. In clinical observation, many cardiovascular diseases (e.g., myocardial infarction, stroke, and arrhythmia) have shown to be closely associated with circadian rhythms (Aronow & Ahn, 2003; Yang et al., 2022). With regard to endogenous rhythms in relation to mortality, sudden cardiac death (SCD) has been mostly studied. Those studies demonstrated that the occurrence of death increases at certain times of the day (Chen & Yang, 2015; Delisle et al., 2021). However, to date, endogenous patterns in relation to mortality of other diseases have rarely been investigated. Whether there is a circadian distribution in overall mortality has yet to be determined. In clinical practice, nurse is the front line of monitoring and treatment for patients, especially for those in intensive care unit (ICU). One study pointed out the importance of research on circadian rhythm and formulated the nursing protocol of identifying circadian health disorders in ICU and hospitalized patients (Padilla‐Martínez et al., 2021). For example, by well understanding the circadian pattern of death, which is the most severe clinical consequence, it would be useful to develop nursing interventions to lower the risk of mortality. Thus herein, we elucidated circadian patterns of mortality among hospitalized patients, with the hypothesis that circadian pattern of in‐hospital mortality is similar to that of SCD.

## 2 METHODS

### 2.1 Study subjects

A retrospective, single institution (Sichuan provincial hospital) registry that included 3300 consecutive deaths between June 2014 and July 2017 was used in this study. The Ethics Committee of our hospital approved the study protocol. Information for the precise time and circumstances of death was reviewed and gathered from medical records. Other information such as demographics and clinical characteristics (e.g., comorbid diseases, resuscitation, the period of time elapsed between the beginning of symptoms and death, and drug therapy) was also collected. Adult deceased patients (age ≥18 years old) with death certificates were included. Those with unclear onset time of death and those corresponding to violent deaths (homicides, suicides, or accidents) were excluded.

### 2.2 Definition

Each day was divided into 24 equal parts of 1 h each. Patients were grouped according to the time of death. Given that there is no standard time frame to distinct the day and the night, we referenced the rule used by the previous report (Bae et al., 2010) and defined the day time ranging from 06:00 to 18:00 and the night ranging from 18:00 to 06:00. SCD was defined as death from cardiac disease occurring less than or equal to 1 h after the onset of symptoms (Muller et al., 1987).

### 2.3 Statistics

In the analysis, demographic and clinical characteristics were first described. To identify whether the circadian pattern of the incidence of hospitalized death mimics that of SCD, we compared the circadian distribution of SCD with that of non‐SCD in our cohort. For further verification, we extracted data of SCD from two previous studies (Muller et al., 1987; Thakur et al., 1996) and compared their pattern of SCD with our result.

SPSS 23.0 was applied for statistical analysis. The Kolmogorov–Smirnov test was used to compare the difference of the circadian variation of death between different groups. Harmonic regression as a method for analysis of diurnal rhythms was previously applied to monitor blood pressure and angina attack rate (Gaffney et al., 1993). In our study to quantify the periodic structure of the frequency of occurrence of death, harmonic regression model was fitted to the published data (Muller et al., 1987). Herein, we implemented Harmonic Analysis of Time Series (HANTS) in Matlab (source codes at http://gdsc.nlr.nl/gdsc/en/tools/hants) (Abouali, 2021) to estimate the magnitude and period of death occurrence. The core algorithm of HANTS is the least square method and Fourier transform, which are used for the decomposition and reconstruction of the death curve, aiming at linking spatial distribution and temporal change. The period of oscillation was 24 h. Results were considered statistically significant at *p* < 0.05.

## 3 RESULTS

A total of 3300 cases were included in the present study. 2094 cases (63.4%) were men, representing a male–female ratio of 1.7:1. The median age was 73 years old. 1540 (46.7%) cases died in the ICU, and 1113 (33.7%) were surgical patients. With regard to diagnosis at admission, diseases of the circulatory system, respiratory system, and central nervous system (CNS) accounted for 13.1%, 32.0%, and 15.0%, respectively. A total of 371 (11.2%) cases were related to SCD. On the whole, more deaths occurred during daytime than at night (54.2% vs. 45.8%; Table 1).

**TABLE 1:** Demographic and clinical characteristics of in‐hospital adult mortality.

	*N* = 3300
Male	2094 (63.5)
Age (median, IRQ, years)	73 (59–82)
18–44	285 (8.6)
45–64	826 (25.0)
≥65	2189 (66.3)
Hospital stay (median, IRQ, days)	8.5 (2–19)
ICU patients	1540 (46.7)
Surgical Pt	1113 (33.7)
Principal diagnosis at admission	
Diseases of the circulatory system	433 (13.1)
Diseases of the respiratory system	1055 (32.0)
Diseases of CNS	631 (19.1)
Sudden cardiac death	371 (11.2%)
Interval from onset of symptoms to death (median, IRQ, minutes)	34 (23–55)
Death time	
Day (6:00 A.M. to 5:59 P.M.)	1787 (54.2)
Sudden cardiac death	199 (6.0)
Non‐sudden cardiac death	1588 (48.1)
Night (6:00 P.M. to 5:59 A.M.)	1513 (45.9)
Sudden cardiac death	172 (5.2)
Non‐sudden cardiac death	1341 (40.6)

Abbreviation: CNS, central nervous system.

**FIGURE 1:**
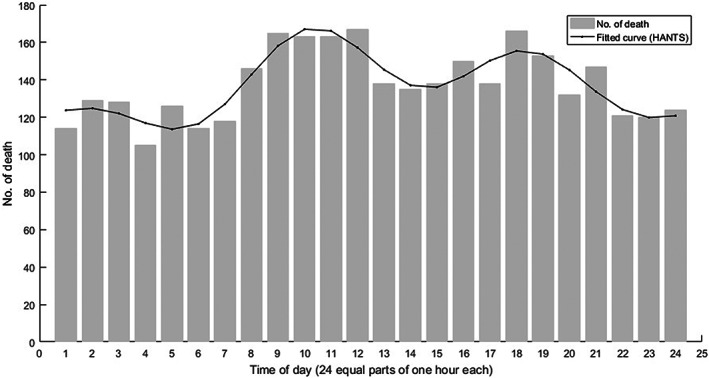
Circadian variation in the incidence of hospitalized death. The fitted curve is shown in black (*p* < 0.01, *n* = 3300).

Figure 1 presents a bimodal distribution of the occurrence of death during a whole day for hospitalized patients. Circadian rhythm was evident with a statistically significant difference (*p* < 0.01). Peaks were detected between 07:00–12:00 and 15:00–20:00, which showed a 21.5% and 13.1% increase above the average, respectively. The lowest death incidence was at 04:00–05:00 with 17.4% below the average.

**FIGURE 2:**
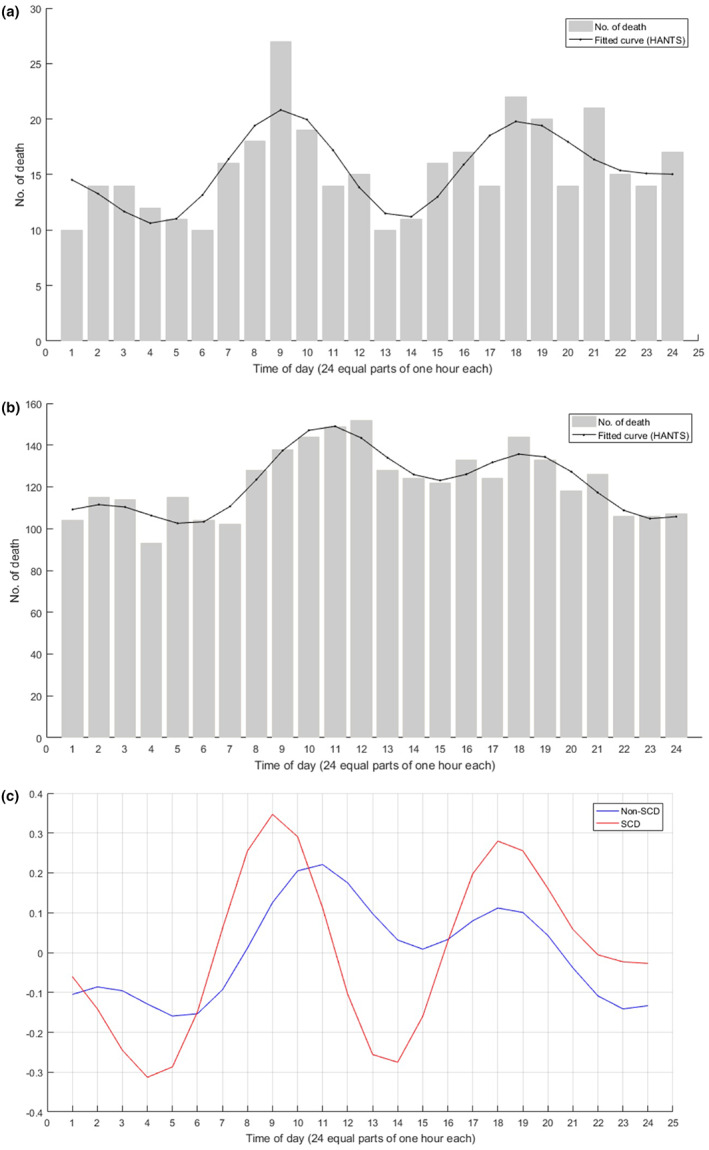
(a) Circadian variation in the frequency of sudden cardiac death (SCD). The fitted curve is shown in black (*p* < 0.01, *n* = 371). (b) Circadian variation in the frequency of non‐SCD. The fitted curve is shown in black (*p* < 0.01, *n* = 2929). (c) Circadian variation based on fitted curve among SCD and non‐SCD. The circadian curve was calculated by (fitted curve value − average fitted curve value)/average fitted curve value.

Figure 2 demonstrates a similar bimodal distribution of the occurrence of death during a whole day for both SCD and non‐SCD groups (Kolmogorov–Smirnov *Z* 0.812, *p* = 0.525). Circadian rhythm was evident with a statistically significant difference (*p* < 0.01; Figure 2c). Peaks for SCD were detected between 06:00–12:00 and 15:00–20:00, which showed 34.7% and 28.0% increase above the average, respectively (Figure 2a). Peaks for non‐SCD were detected between 07:00–13:00 and 15:00–20:00, which showed a 22.1% and 11.2% increase above the average, respectively (Figure 2b). The lowest death incidence of SCD was at 04:00–05:00 and 13:00–14:00 with 31.3% and 27.5% below the average, respectively.

**FIGURE 3:**
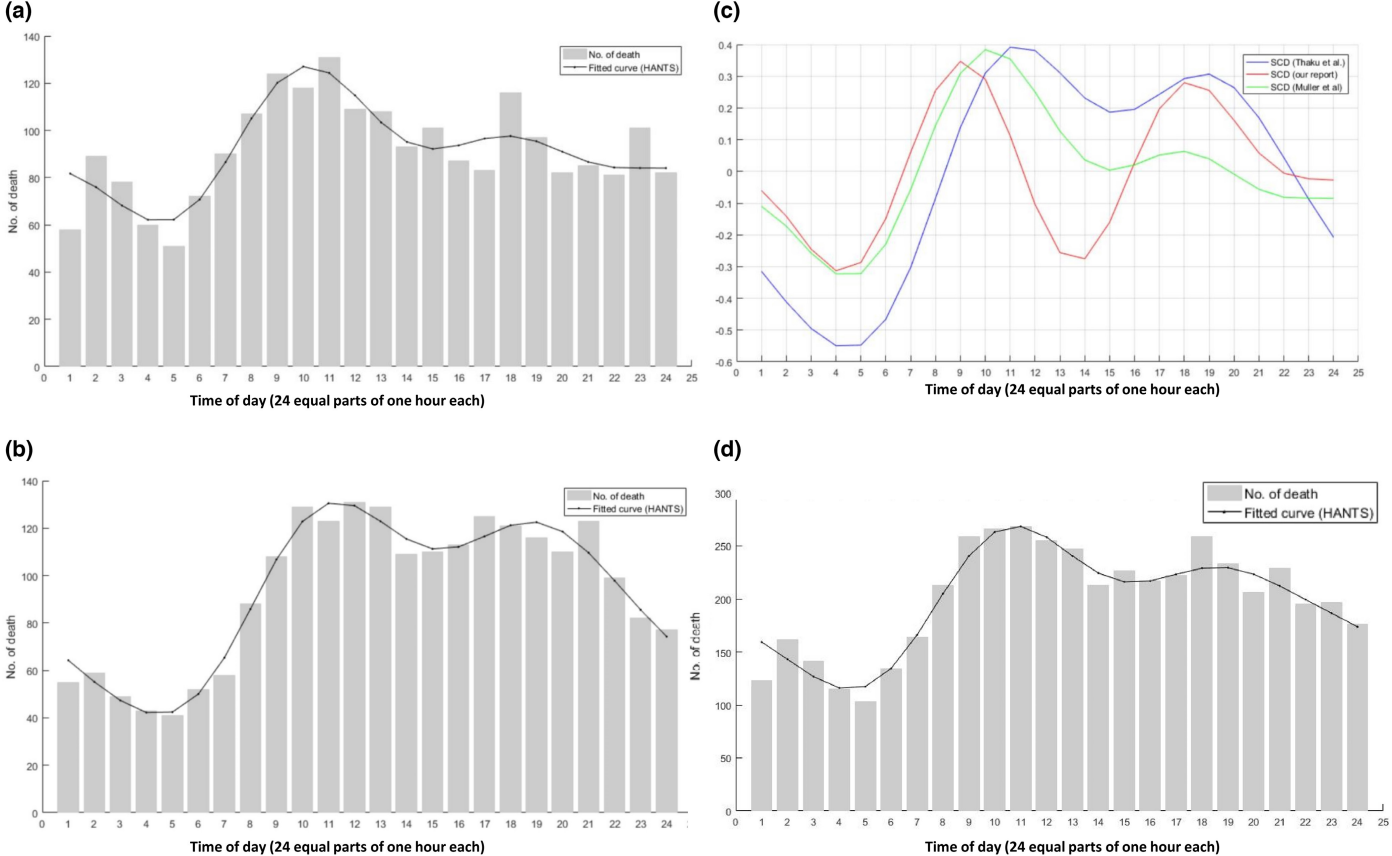
(a) Circadian variation in the frequency of sudden cardiac death (SCD) redrawn from Muller et al. The fitted curve is shown in black (*p* < 0.01, *n* = 2203). (b) Circadian variation in the frequency of SCD redrawn from Thaku et al. The fitted curve is shown in black (*p* < 0.01, *n* = 2250). (c) Circadian variation based on fitted curve of our SCD and previous reports. The circadian curve was calculated by (fitted curve value − average fitted curve value)/average fitted curve value. (d) Circadian variation in the frequency of SCD by integrating the existing data (Muller's, Thaku's and ours).

Figure 3 depicts the bimodal distribution of the occurrence of SCD during a whole day comparing previous reports (Figure 3a,b) with our results. It was noted the second peak in Muller's report (Muller et al., 1987) was flattened (Figure 3a). An abrupt increase in death incidence was observed after 04:00 and continued until 9:00–12:00. The bottom of death incidence of SCD was primarily located at 03:00–05:00. The distribution of the occurrence of SCD in our results showed no statistical difference compared with the findings of Muller et al. (Kolmogorov–Smirnov *Z* 0.764, *p* = 0.603) (Figure 3c). By contrast, a difference was noted when compared with the results of Thaku et al. (Kolmogorov–Smirnov *Z* 1.874, *p* = 0.002) (Figure 3c). By integrating the existing data (Muller et al., Thaku et al. and ours), bimodal distribution of the occurrence of SCD was also observed (Figure 3d). In addition, subgroup (sex, age, and primary diagnosis) analysis regarding the distribution of the occurrence of death in a whole day was further conducted and shown in Figure S1.

## 4 DISCUSSION

Vascular events in previous reports which show an excess of death between 06:00 and noon are in line with our in‐hospital mortality pattern, indicating the possible existence of similar underlying mechanism related to in‐hospital mortality, although the physiological mechanism of circadian variation is not well understood and remains speculative. Those events include myocardial angina, acute myocardial infarction, cardiac arrest, arrhythmias (de Rueda et al., 2022; Muller et al., 1989; Xin et al., 2022), deep venous thrombosis, pulmonary thromboembolism (Bilora et al., 2001; Colantonio et al., 1989; Damnjanović, 2018; Gallerani et al., 1994), and stroke (ischemic and haemorrhagic strokes and transient ischemic attacks) (Argentino et al., 1990; Elliott, 1998; Yang et al., 2022). The implication for nursing care is that strengthening monitor for patients with those disorders at certain time may be needed. For example, myocardial infarction was thought to be influenced by increases in blood pressure, pulse rate, and platelet aggregability in the morning hours, (Tofler et al., 1987). Intensified monitoring and prompt intervention are necessary to avoid unexpected adverse events.

The present study explored hospitalized death, which could be caused by a variety of diseases or complications. The purpose of the study was to discover the circadian rhythm of overall hospitalized mortality from a clinical angle, which is supportive of the assumption that a number of fatal events intrigue death under a circadian background. Thus, we did not intend to categorize the deceased patients into several subgroups with putative death causes. Instead we chose SCD, which has been intensively investigated previously, as the linking node bridging the comparison between our results (SCD vs. non‐SCD) and others (SCD in our study vs. SCD in the literature). In the present study, non‐SCD was similar to SCD with respect to circadian patterns of death incidence, although a small number of deaths may be unrelated to the same circadian pattern. For instance, in one study, unexpected deaths following major surgical procedures revealed high incidence at night (13 deaths at night vs. 5 during day) (Rosenberg et al., 1992). In hospital, cardiovascular factors are assumed to play a critical role for circadian rhythm of death. A circadian pattern of death was also observed in patients with congestive heart failure with the primary peak occurred between 06:00 and 12:00 (Aronow & Ahn, 2003). In contrast to cardiovascular events, other events such as respiratory failure could be less likely fatal because a ventilator is always accessible to maintain life. Note that a certain number of patients with cardiac problems may be included in the non‐SCD group, which may potentially affect the circadian structure of death incidence. Some of those patients could die of cardiac events and did not pertain to SCD group according to our definition. One study reported that patients with prevalent haemodialysis had an excess of morning deaths, and 24.8% more deaths occurred from 4:00 to 12:00 (Tislér et al., 2008), which is consistent with our results. In that study, death was not limited to certain causes.

The lowest incidence of death at 04:00–05:00 among SCD is of interest. By reviewing the literature, some reports validated the phenomenon in SCD (Aronow & Ahn, 2003; Muller et al., 1987; Thakur et al., 1996). Some other studies also identified an early morning nadir of sudden cardiac arrest between 12 A.M. and 6 A.M. (Ramireddy & Chugh, 2021). One possible explanation is circadian pattern of physiological function may be relate to and affect the timing of death. For instance, blood pressure is usually lower in sleep than in wakefulness and has a characteristic surge after awakening, paralleling with the daily pattern of relative high adverse cardiovascular events (Ohkubo et al., 1997; Taylor et al., 2015). This phenomenon has given rise to an increasing interest in underlying mechanism of low mortality rate at the hours before morning, which is beneficial to assess interventions to reduce the risk of morning events. For example, individual drug administration for blood pressure management according to physiological rhythm, creating comfortable environment of sleep to avoid the disintegration of sleep structure and other relevant measures would benefit for patient recovery.

One study found rotating shift nurses behaved worse perception in organizational and work environmental factors (Gómez‐García et al., 2016). In the context, another consideration that whether factors such as night shift and rotating, being distracted/fatigue after long‐hour work, overloaded, working environment play a significant role on circadian rhythm of in‐hospital mortality need further investigation to address.

### 4.1 Limitation

There are several limitations. First, a retrospective design could cause biases (e.g., selective bias). Second, forensic autopsies were not performed for the overwhelming majority of the deceased, so the cause of death of some patients cannot be certificated. Third, given that the results were from a single hospital, any extrapolation of these results to the general hospitalized patients must be cautious.

## 5 CONCLUSION

In summary, this work is the first study to document a circadian pattern of death in hospitalized patients. Our findings confirmed the prominent circadian pattern in SCD and demonstrated a similar pattern in hospitalized death, indicating a similar mechanism shared. At the same time, our study stressed the importance for preventing in‐hospital mortality by introducing corresponding nursing care at certain time of the day.

## AUTHOR CONTRIBUTIONS

Miao Fang: investigation, formal analysis, original draft writing. Shunju Xiang: investigation, original draft writing. Xiaoqiang Xiao: investigation, formal analysis. Qianning Mo: methodology, writing – review & editing. Yang Si: conceptualization methodology, validation, writing – original draft, writing – review & editing.

## FUNDING INFORMATION

None.

## CONFLICT OF INTEREST STATEMENT

None.

## ETHICS STATEMENT

The Ethics Committee of our hospital approved the study protocol.

## Supporting information


Figure S1.

## Data Availability

The de‐identified data that used in this study are available from the corresponding author upon reasonable request.
