# Supplementary figures and images for: Circadian aspects of mortality in hospitalized patients: A retrospective observation from a large cohort
Source: Nurs Open. 2023 Mar 8;10(7):4630–6. doi: 10.1002/nop2.1711 (PMC10277412; doi:10.1002/nop2.1711)

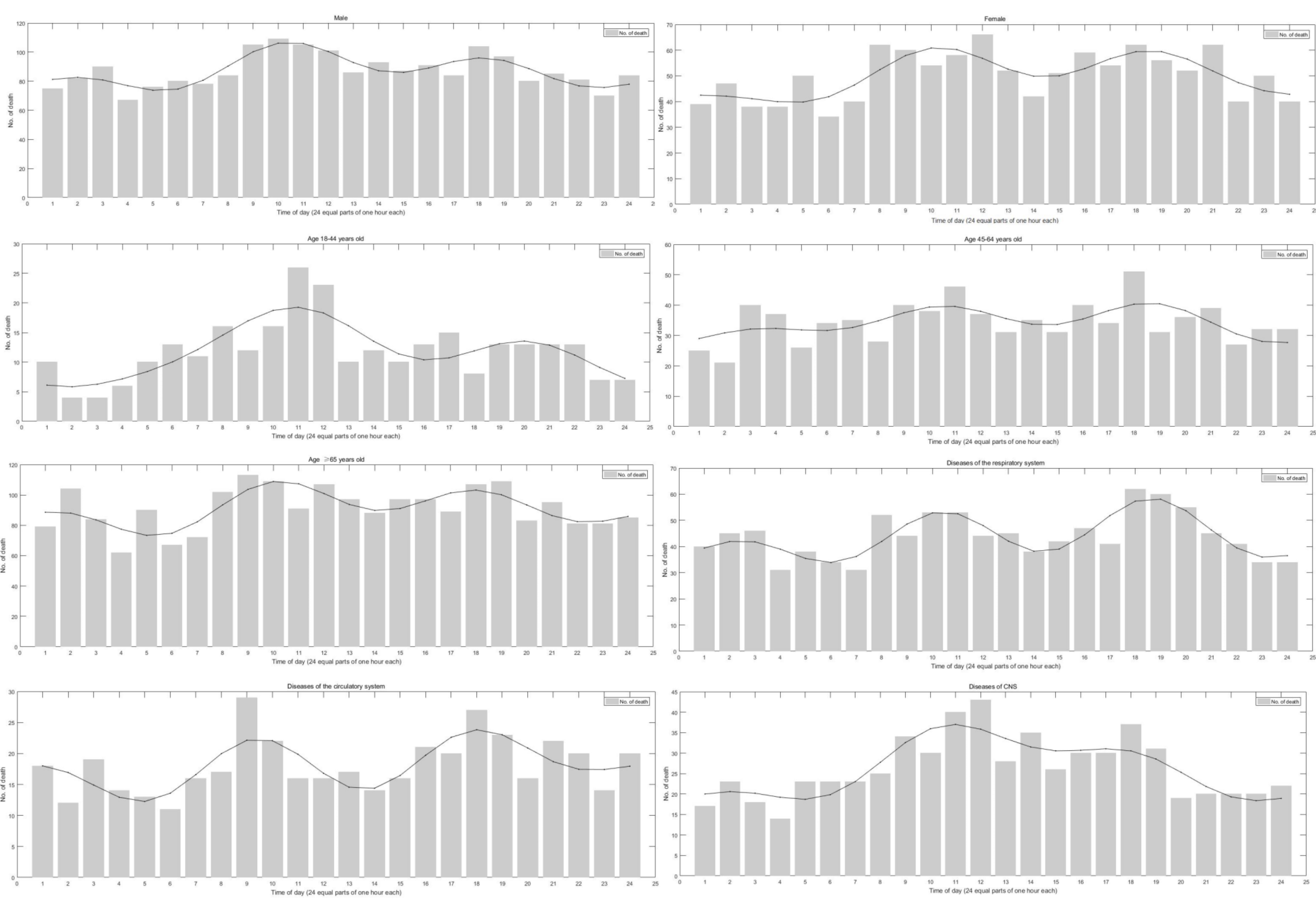

Supplement: Supplementary file 1 — Figure S1. [file NOP2-10-4630-s001.jpg]
